# Y-Stent Rescue Technique for Failed Thrombectomy in Patients With Large Vessel Occlusion: A Case Series and Pooled Analysis

**DOI:** 10.3389/fneur.2020.00924

**Published:** 2020-08-27

**Authors:** Zifu Li, Peng Liu, Lei Zhang, Yongwei Zhang, Yibin Fang, Pengfei Xing, Qinghai Huang, Pengfei Yang, Jianmin Liu

**Affiliations:** Neurosurgery Department, Changhai Hospital, Second Military Medical University, Shanghai, China

**Keywords:** thrombectomy, stroke, artery, stent retriever, occlusion

## Abstract

**Objective:** Y-stent thrombectomy is a recent rescue technique for failed thrombectomy in patients with emergent large vessel occlusion. We presented case series of using Y-stent rescue technique at different sites and investigate its feasibility and safety through pooled analysis of collected case report or series.

**Methods:** Twenty-eight cases were screened from stroke databank who underwent thrombectomy between January 2015 and June 2019. Clinical, procedural, and follow-up data were investigated and pooled analysis of published literature was analyzed.

**Results:** The occlusion sites include carotid terminus in 14 patients; siphon segment in 3; middle cerebral artery (MCA) in 4; basilar terminus in 7. The overall recanalization rate reached 85.7% (arterial occlusive lesion score 2–3); and final reperfusion rate 85.7% (modified Thrombolysis in Cerebral Infarction 2b−3). After literature review, totally, 52 cases were included. Good clinical outcome was achieved in 26 (50%) and mortality in 7 (17.3%). There is no significant difference on the SAH complication at different sites. Literature review shows no difference between each site in the reperfusion and complication rate.

**Conclusion:** Our case series results suggest that high recanalization rate can be effectively achieved with Y-stent rescue technique for patients with refractory emergent large vessel occlusion. The safety of using this technique at different sites needs further investigation for patients.

## Introduction

Mechanical thrombectomy has been the first-line therapy for acute ischemic stroke secondary to emergent large vessel occlusion (ELVO). By recanalizing the occluded artery and restoring blood flow, clinical outcome is remarkably improved (1, 2). However, despite the availability of large-bore aspiration catheter, second-generation stent retriever and balloon guiding catheter, there is at least 10% of patients who fail to recanalize the occluded artery (3–6).

Thrombectomy for the refractory occlusions is challenging because the occlusion is mostly caused by cardiogenic clots (7). Y-stent rescue thrombectomy technique was reported to be a feasible approach by deploying two stent retrievers separately in two separate branches to retrieve the clot (8, 9). The two stent retrievers coordinates together to grip the clot. The technique has been used in refractory cases for occlusion at middle cerebral artery (MCA), basilar artery, and internal carotid artery (ICA) (8–11). However, feasibility of this technique remains unknown owing to limited cases. Therefore, analysis of our prospectively collected data, together with literature review on procedural results, complications, and outcome, was conducted in patients who underwent thrombectomy with the rescue technique.

## Methods

### Patient Selection

All patients were retrospectively reviewed from our prospectively collected database of ELVO who accepted mechanical thrombectomy between January 2015 and March 2019. All raw data were initially collected from the case record form designed for thrombectomy patients. Twenty-eight patients were eligible for the observational study who received Y-stent thrombectomy. This study was approved by the hospital review board.

### Y-Stent Thrombectomy Procedure

**Figure 1:**
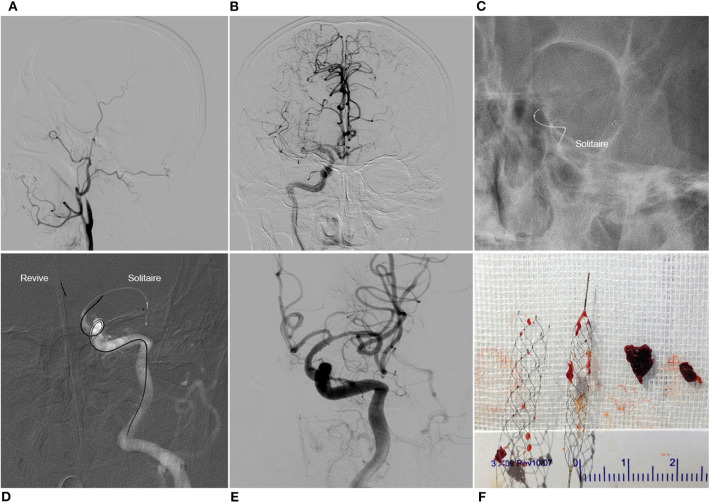
Y-stent thrombectomy technique in a patient with left carotid terminus occlusion. Left common carotid artery angiogram showed the left internal carotid artery occlusion **(A)**. Contralateral angiogram showed pial collaterals from ipsilateral anterior cerebral artery and the existence of the anterior communicating artery, and the chronic occlusion of right middle cerebral artery **(B)**. Solitaire FR 6 × 30 mm was deployed in the left middle cerebral artery **(C)**. Revive SE was then deployed in the left anterior cerebral artery **(D)**. With a single pass of Y-stent thrombectomy, complete recanalization was achieved **(E)**. Clots were captured by the two stent retrievers **(F)**.

**Figure 2:**
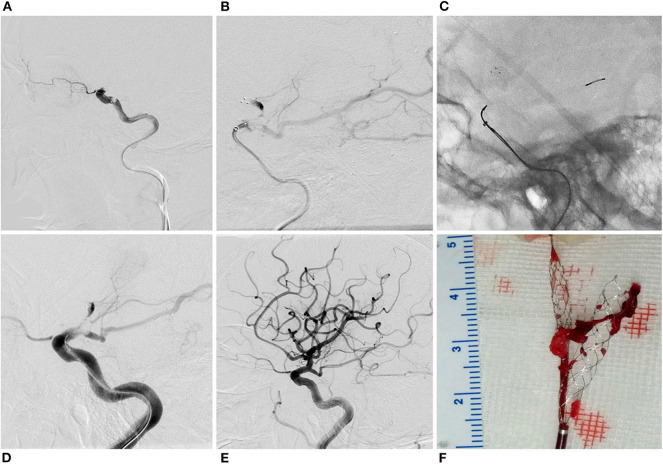
Y-stent technique in a patient with carotid siphon occlusion. Right internal carotid artery angiogram showed carotid siphon occlusion **(A)**. Solitaire FR 6 × 30 mm was deployed in the right middle cerebral artery **(B)**. Revive SE was then deployed in the posterior communicating artery **(C)**. Angiogram showed antegrade flow after stent retriever deployment **(D)**. Complete recanalization was obtained with a single pass of Y-stent thrombectomy **(E)**. Clots were gripped by the two stent retrievers **(F)**.

**Figure 3:**
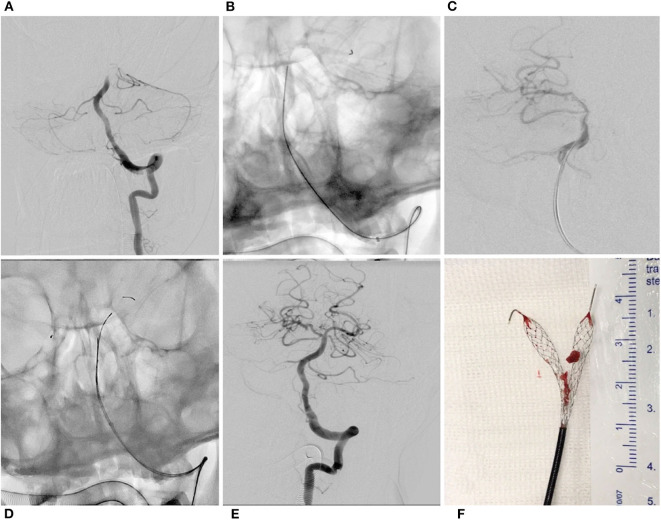
Y-stent thrombectomy technique in a patient with basilar terminus occlusion. Left vertebral artery angiogram showed basilar terminus occlusion **(A)**. Revive SE was unsheathed in the left posterior cerebral artery **(B)**. Angiogram showed bypass flow after stent retriever deployment in the right posterior cerebral artery **(C)**. Revive SE was then deployed in the left posterior cerebral artery **(D)**. Complete recanalization was obtained with a single pass of Y-stent thrombectomy **(E)**. Clots were gripped by the double Revive SEs **(F)**.

Mechanical thrombectomy was first performed using a direct aspiration first pass technique (ADAPT) or solumbra technique. The failure of standard technique was defined as failure of recanalizing the target vessel using solumbra technique for at least two times or together with ADAPT technique for at least three times. Y-stent thrombectomy would be attempted after failure of standard technique. Two microcatheters (Rebar 18 or headway 21) supported by 0.014-in. microwire (Traxcess) were separately advanced across the occlusion and positioned in side branches. Vessel diameter of side branches was measured after angiogram performed via intermediate catheter or superselective angiogram through microcatheter. Stent retrievers including Solitaire FR (ev3 Inc, Plymouth, MN, USA),Revive SE (Codman & Shurtleff, Raynham, MA, USA), and Aperio (Acandis, Germany) were deployed using kissing stent technique. If the intermediate catheter (Navien or sofia) cannot hold two microcatheters, one microcatheter was removed after deployment of the first stent retriever, and the second microcatheter supported by microwire was subsequently advanced without crossing the first stent retriever strut and then the second stent retriever was deployed. For carotid terminus occlusion, the stent retrievers were separately positioned in MCA and anterior cerebral artery (ACA) (Figure 1), carotid siphon segment occlusion in the posterior communicating artery (PcomA) of diameter ≥1.5 mm and ICA (Figure 2), and MCA occlusion in superior and inferior trunk and for basilar terminus occlusion positioned in bilateral posterior cerebral artery (Figure 3). Aspiration via the intermediate and guiding catheters was routinely applied during the retrieval procedure. Angiographic runs were performed immediately after the procedure to assess the recanalization of the occluded bifurcation. If side branch was occluded with patent bifurcation, thrombectomy with single stent retriever technique or ADAPT would be subsequently performed.

### Outcome Analysis

Successful reperfusion result was defined as the modified thrombolysis in cerebral infarction (mTICI) 2b−3. Successful recanalization result was defined as the Arterial Occlusive Lesion (AOL) score 2–3. The Y-stent AOL score was defined as the immediate recanalization score with Y-stent technique. Good clinical outcome was defined as modified Rankin Score (mRS) 0–2 at 3-month follow-up.

### Literature Review

A search of the PubMed database identified all studies between 2012 and 2019 including case reports and series on the application of double stent retrievers. The key word includes “thrombectomy,” “Y-stent,” “dual stent,” “Y-configuration,” “rescue technique,” and “stroke.” The related data were extracted from the eligible studies.

### Statistical Analysis

Descriptive analysis included percentages for categorical variables and median ± SD or mean for continuous variables. Procedural and outcome results were analyzed using the χ^2^ test or Fisher exact test for categorical variables. Statistical analyses were performed using SPSS, version 20.0 (Chicago, IL, USA). *P* < 0.05 was considered significant.

## Results

### Patient Baseline

A total of 637 patients accepted mechanical thrombectomy during the research, of which 28 patients were eligible as failed thrombectomy, accounting for 4.4%. The occlusion sites included the carotid terminus in 14 patients, carotid siphon in 3, MCA bifurcation in 4, and basilar terminus in 7. Among the 28 patients, embolic ELVO was diagnosed in 24 patients including 14 with cardiogenic strokes associated with atrial fibrillation or cardiovascular surgery, 10 with cryptogenic etiology, and atherosclerotic occlusion in 4 patients including 3 with tandem occlusion resulting from proximal atherosclerotic occlusion and 1 with atherosclerotic occlusion of the carotid terminus. The baseline characteristics of the eligible patients are presented in Table 1.

**Table 1:** Characteristic of patients with refractory emergent large vessel occlusion.

**Characteristic**	***N* = 28**
Age, mean (SD), years	66.9 (9.6)
Male, no./total (%)	18/28 (64.3)
Occlusion etiology, no./total (%)	
Large artery atherosclerosis	4/28 (14.3)
Cardiogenic	14/28 (50.0)
Cryptogenic embolism	10/28 (35.7)
Site of occlusion, no./total (%)	
Carotid bifurcation	14/28 (50.0)
Carotid siphon	3/28 (10.7)
MCA bifurcation	4/28 (14.3)
Basilar terminus	7/28 (25.0)
Baseline NIHSS score, mean (SD)	21 ± 8.0
NIHSS score at discharge, mean (SD)	17.3 ± 12.4
Alteplase bridging therapy, no./total (%)	6/28 (21.4)
Door to needle time, median (range)—min	36 (26–68)
Door to groin puncture, median (range)—min	83 (23–450)
Groin puncture to mTICI 2b or 3, median (range)—min	111 (44–309)
Passes before Y-stent thrombectomy, median (range)	4 (2–8)
Procedural complications, no./total (%)	
SAH	3/28 (10.7)
Dissection	1/28 (3.6)
Vasospasm	1/28 (3.6)
Downstream embolism	1/28 (3.6)
Recanalization results, no./total (%)	
AOL	24/28 (85.7)
mTICI 2b−3	24/28 (85.7)
mRS 0–2 at 3 months	12/28 (42.9)
Mortality	6/28 (21.4)

*NIHSS, National Institute of Health Stroke Scale; ICA-PComA, internal carotid artery–posterior communicating artery; MCA, middle cerebral artery; IQR, interquartile range; mTICI, modified thrombolysis in cerebral infarction score*.

### Procedural Results

The stent retrievers before Y-stent technique were used with Solitaire FR in 19 patients, Revive SE in 6, Trevo followed by Solitaire FR in 1, and Revive SE followed by Solitaire FR in 2. Aspiration technique was initially used in 10 cases. The median number of device passes is 4 times (range, 2–8). The Y-stent technique with Solitaire FR plus Revive SE was used in 14 patients, double Solitaires FR in 10,double Revive SEs in two, Solitaire FR plus Aperio in one patient, and Trevo plus Solitaire in one.

The Y-stent technique was successfully implemented in 27 patients, and 1 failed because the second stent retriever was released through the mesh of the first deployed stent retriever. Successful rescue reperfusion (mTICI 2b−3) and immediate successful recanalization after using Y-stent technique (AOL 2-3) were respectively obtained in 24 (85.7%) patients. The occluded vessel was reanalyzed with the technique after one pass in 21 (87.5%), two passes in two patients, and three passes in one. No significant difference was observed in the recanalization rate between the four sites; the rescue technique tends to bring higher recanalization rate at the two sites of basilar terminus (100%) and carotid terminus (92.9%) than carotid siphon (33.3%). The embolism fragments retrieved from the three patients with carotid siphon occlusion was specially inspected, and we found the clot was white and as elastic as rubber. According to the stroke etiology, the successful recanalization is similar between embolic occlusion and atherosclerotic occlusion (21/24, 87.5 vs. 3/4, 75%; *p* = 1). However, despite no significance observed, the reperfusion rate for atherosclerotic occlusion tends to be lower than embolic occlusion (2/4, 50 vs. 22/24, 91.7%; *p* = 0.152). The procedural results are presented in Table 2.

**Table 2:** Procedural results of Y-stent technique for large vessel occlusion at different sites.

	**Total**	**Carotid terminus**	**Carotid siphon**	**MCA bifurcation**	**Basilar terminus**
	28	14	3	4	7
Angiographic results					
AOL 2 or 3	24 (85.7%)	13 (92.9%)	1 (33.3%)	3 (75%)	7 (100%)
mTICI 2b or 3	24 (85.7%)	11 (78.6%)	3 (100%)	3 (75%)	7 (100%)
Complications					
PH	1 (3.6%)	1 (7.1%)	0	0	0
Distal embolism	1 (3.6%)	1 (7.1%)	0	0	0
SAH	3 (12.5%)	1 (7.1%)	0	2 (50.0%)	0
Dissection	1 (3.6%)	0	0	0	1 (14.3%)
Vasospasm	1 (3.6%)	1 (7.1%)	0	0	0
mRS 0–2	12 (42.9%)	5 (35.7%)	1 (33.3%)	2 (50%)	4 (57.1%)
Mortality	6 (21.4%)	2 (14.3%)	2 (66.7%)	0	2 (28.6%)

*AOL, arterial occlusion lesion; mTICI, modified thrombolysis in cerebral infarction; PH, parenchymal hematoma; SAH, subarachnoid hemorrhage; mRS, modified Rankin Score; MCA, middle cerebral artery*.

### Complications

The procedural complication of subarachnoid hemorrhage (SAH) occurred in 3 patients including 2 (50.0%) of 4 patients with MCA occlusion and 1 (7.1%) of 14 patients with carotid terminus occlusion, and none with carotid siphon and basilar terminus occlusion. The procedural SAH rate at the MCA occlusion site tends to be higher than non-MCA group including the remaining three sites (50.0 vs. 4.2%; *p* = 0.061), although there is no statistical significance. Parenchymal hematoma occurred in one patient with carotid terminus occlusion as a result of hemorrhagic transformation, and craniectomy and hematoma evacuation were performed. The patient finally died of pneumonia at 2 weeks. Downstream embolism occurred in one patient (case 13) with atherosclerotic carotid terminus occlusion. In the patient, we initially assumed it as embolic occlusion and mistakenly retrieved the clot for four times, resulting in secondary *in situ* thrombus formation and downstream embolism. Vasospasm was observed in one patient with carotid terminus occlusion. After infusion of fasudil via guiding catheter, the vasospasm was relieved. The procedural complication is presented in Table 2.

### Clinical Outcome

At 3-month follow-up, good clinical outcome (mRS 0–2) was achieved in 12 (42.9%) of 28 patients including 5 (35.7%) of 14 patients with carotid terminus occlusion, 2 (50.0%) of 4 patients with MCA bifurcation occlusion, 1 (33.3%) of 3 patients with carotid siphon segment occlusion, and 4 (57.1%) of 7 patients with basilar terminus occlusion. Six patients died including one patient (case 16) who died from massive infarction, one (case 1) from parenchymal hematoma, one (case 17) from aortic dissecting aneurysm who received aortic root replacement in the cardiac surgery department and then experienced embolic stroke, and one (case 27) from pneumonia at 3-month follow-up, and two (case 4 and case 28) from unavailable reasons. The detailed outcome is shown in Table S1.

### Literature Review Results

After literature review, 24 cases with different site occlusions were included for pooled analysis from five published articles (8, 9, 11–14), including 19 cases with MCA, 1 with basilar terminus, and 4 with carotid terminus. Clinical outcome was unavailable in three patients with MCA occlusion and one patient with basilar terminus (9, 12, 13). The detailed procedural results plus ours are presented in Table 3. Although no statistically significant difference was observed in the recanalization, reperfusion, complication, and outcome rate, the complication rate in the MCA occlusion subgroup tends to higher than other subgroups.

**Table 3:** Literature reviews studies on Y-stent technique for large vessel occlusion at different sites.

	**Total**	**Carotid terminus**	**Carotid siphon**	**MCA bifurcation**	**Basilar terminus**
	24	4	0	19	1
Angiographic results					
mTICI 2b or 3	21 (87.5%)	3 (75%)	0	17 (89.5%)	1 (100%)
Procedural complications					
SAH	1 (4.2%)	0	0	1 (5.3%)	0
Dissection	0	0	0	0	0
Vasospasm	5 (20.8%)	0	0	5 (26.3%)	0
mRS 0–2	13 (54.2%)	1 (25%)	0	12 (63.2%)^*^	^*^
Mortality	2 (8.3%)	2 (50.0%)	0	0^*^	^*^

*AOL, arterial occlusion lesion; mTICI, modified thrombolysis in cerebral infarction; SAH, subarachnoid hemorrhage; mRS, modified Rankin Score; MCA, middle cerebral artery*.

* *Data were not available in one reported case*.

## Discussion

The case series demonstrates the application of Y-stent rescue technique at different bifurcation sites for refractory ELVO. The recanalization (AOL 2–3) and reperfusion rate (mTICI 2b−3) separately reached 85.7%. Among the four sites, the rescue technique tends to have higher recanalization rate at the two sites of carotid (92.9%) and basilar terminus (100%) than carotid siphon segment (33.3%). Despite no significance, the reperfusion rate tends to be lower for atherosclerotic occlusion than embolic occlusion (50 vs. 91.7%, *p* = 0.152). After using Y-stent technique, 42.9% patients achieved good clinical outcome. Although no statistically significant difference was observed in the recanalization, safety and good clinical outcome rate between the four sites after the literature review, SAH occurred more often in patients with MCA bifurcation occlusion (50.0%) than other three sites.

Several articles showed more thrombectomy passes associated with lower recanalization rate and poor outcome (15, 16). Especially, when the number of thrombectomy times increases from 2 to 3, there is an obvious fall of recanalization rate (15). Therefore, combining our experience, we defined refractory thrombectomies as failure of recanalizing the target vessel using solumbra technique for at least two times or together with ADAPT technique for at least three times. Even so, the pre-rescue attempts are still based on operator's decision influenced by several factors, including clot load/length of the occluded arterial segment, size and composition of the already retrieved clot fragments, and the amount of clot extension into the bifurcation limbs.

For bifurcation occlusion, the main body of the clot usually exists in the branch with a bigger diameter and less tortuosity. For example, the main body of the clot in carotid T occlusions is usually in the MCA. Generally, we tend to use Solitaire 6 × 30 mm which is bigger. If it fails, we use the Y-stent technique. As the ACA has a long tortuous section, we tend to use Revive, which is non-detachable, as opposed to Solitaire AB, which has the risk of breaking in the potential detachment point (17). In addition, Revive is recommended to be used in a blood vessel of 1.5 mm. For the carotid T occlusion in this series, we used Solitaire in MCA and Revive in ACA with the intention to maximize the use of the feature of different devices. Revive has smaller cells to stop clots from migrating whereas Solitaire AB has bigger cells to catch clots in the MCA. Meanwhile, the open cell and split design of Solitaire can minimize intimal injury. For MCA bifurcation occlusion, where the two branches are similar in size and less tortuous, the choice of the stent retriever is less important. In this case series, the Y-stent technique (one Solitaire and one Revive) was applied after both failed. Asadi et al. (9) also reported the application of Trevo ProVue and EmboTrap for the Y-stent thrombectomy technique in a patient with MCA bifurcation occlusion. The visible stent retriever has the advantage of displaying the position of the stent and interaction between the two stent retrievers (12).

Several published reports have described the Y-stent rescue technique for MCA and carotid terminus occlusion (8, 9). Application at other sites were not reported. In our center, we present the application at four different sites including the basilar terminus, carotid siphon segment, MCA bifurcation, and carotid terminus. The technique application was determined by many factors including artery diameter, vasculature tortuosity, and available stent retrievers. Therefore, for carotid siphon segment occlusion with fetal PComA or with diameter ≥1.5 mm, the technique was also attempted. This technique was not suitable for ACA bifurcation occlusion with one dominant A1 because of the small caliber and the tortuous vasculature.

The situation would be desperate if aspiration with large bore aspiration catheter or standard stent retriever thrombectomy plus aspiration was performed for several times without recanalization of target vessel. Clots, especially cardiogenic fibrin-rich clots, are easily stuck in the bifurcation branches and can be squeezed into the other branch during the retriever procedure, and difficult to be aspirated for aspiration technique (18, 19). Many options, especially routinely used stenting technique, can be selected for refractory ELVO (20). The rescue stenting technique has disadvantages for bifurcation occlusion. Stenting at bifurcation may lead to occlusion of branch artery occlusion. Moreover, thrombus at the occluded segment may be squeezed into the stent retriever after release and cause subsequent in-stent occlusion. Y-stent technique has its advantage of simultaneously retrieving the clot by fully engaging the clot and using two stent retrievers to grip the clot together with the intermediate catheter.

With respect to the etiology in our case series, the situation mostly encountered is embolic ELVO, especially cardiogenic. The recanalization rate is similar for atherosclerotic and embolic occlusion (75 vs. 87.5%, *p* = 1). However, the reperfusion rate tends to be lower for atherosclerotic occlusion than embolic occlusion (50 vs. 91.7%; *p* = 0.152) despite no statistically significant difference. For atherosclerotic occlusion besides tandem occlusion, the distal clots can be aspirated with aspiration catheter or retrieved with stent retrievers (21). However, multiple thrombectomy attempts would make the clots more elastic and difficult to engage. In the meantime, multiple device passes would damage the endothelial layer and make the atherosclerotic lesion more irritable, and then secondary *in situ* thrombosis would develop (22, 23). After application of Y-stent technique with two stent retrievers, the damage to the vascular wall would be aggravated, which might result in downstream embolism and low reperfusion rate. Therefore, the optimal occlusion type for Y-stent technique application is embolic occlusion rather than atherosclerotic occlusion.

The recanalization and reperfusion rate have no difference between the four sites. However, the recanalization rate tends to be higher at the occlusion sites of carotid (92.9%) and basilar terminus (100%) than carotid siphon (33.3%). Y-stent technique failed in two patients with carotid siphon occlusion and rescue stenting was obligated to be performed. The tendency of low recanalization rate at carotid siphon was observed in our cases series even with Y-stent technique. We considered that the high failure rate at the siphon segment was associated with the clot profile. Embolic clots are easily stuck at bifurcation instead of siphon curve. Our results showed that the clot fragments retrieved from the three patients are more elastic than usual. The successful recanalization and good clinical outcome rates are similar to that in the Klisch's report of using Y-stent technique (8). However, the recanalization rate was higher than that of 78.4% in the reported study that used the variable thrombectomy technique (24), and also higher than that in other studies with stent retrievers (25, 26).

A major concern is endothelial injury and artery displacement when two stent retrievers were used simultaneously, especially released in small caliber arteries. In our case series, after multiple single stent retriever and Y-stent passes, procedural SAH occurred in two patients at MCA bifurcation and one at carotid terminus. The SAH complication rate at the MCA bifurcation is higher than non-MCA sites (50.0 vs. 4.2%; *p* = 0.061). Although the literature review further similarly shows that no significance was observed in the procedural SAH rate, the technique should be cautiously recommended for MCA bifurcation occlusion. When stent retriever was placed in distal M2 segment with a small caliber, retrieving the double stents simultaneously may lead to artery displacement and small perforators rupture. Therefore, partial re-sheathing of the stent retrievers after full deployment is advised at these sites to reduce the resistance of pulling (27). Low SAH complication rates were observed at the site of carotid and basilar terminus. This may be attributed to large vessel caliber and straight vasculature. Although the procedural complication for MCA occlusion was clinically insignificant in our cases, the technique was only used as the last resort to tackle desperate situations.

The overall mortality rate and good clinical outcome is similar to that in other studies, respectively reached 21.4 and 42.9%, compared with 13.6 and 63.6% in the meta-analysis of five randomized trials (1), and 20 and 50% in Klisch's study with double Solitaire technique (8). Comparing with stenting rescue technique, the good clinical outcome seems to be better than that (42.9 vs. 39.6%) in Chang's study and has similar mortality (13.6 vs. 12.5%) (28).

The retrospective study has a few limitations. The population is heterogeneous regarding the locations, different stent retrievers, number of attempts for standard technique, and time to recanalization. Further investigation in more cases at different sites is required to validate its effectiveness and safety. Especially for MCA occlusion, the procedural SAH complication is higher than other sites in our series; more cases were needed to eliminate the patient selection bias. Therefore, despite the high recanalization rate of the rescue technique, the results should not be overinterpreted. The technique potentially results in endothelial damage to arterial wall and is only recommended as the last resort to tackle difficult situation.

## Conclusion

In conclusion, our case series suggest that high recanalization rate can be achieved with Y-stent rescue thrombectomy technique for embolic refractory ELVO. At four different sites, this technique seems to be safer at the sites of carotid and basilar terminus than MCA. Further investigation with large sample size is required to evaluate its effectiveness and safety at different sites.

## Data Availability Statement

The raw data supporting the conclusions of this article will be made available by the authors, without undue reservation.

## Ethics Statement

The studies involving human participants were reviewed and approved by Changhai Hospital Review Board. The patients/participants provided their written informed consent to participate in this study.

## Author Contributions

ZL, PL, PY, and JL participated in the design of this study. ZL and PL drafted the manuscript. ZL performed statistical analysis. PL carried out the collected important background information. LZ and YZ carried out literature search. YF and PX helped the literature search and statistical acquisition. QH, PY, and JL modified the manuscript. All authors read and approved the final manuscript.

## Conflict of Interest

The authors declare that the research was conducted in the absence of any commercial or financial relationships that could be construed as a potential conflict of interest.
