# Supplementary material for: Y-Stent Rescue Technique for Failed Thrombectomy in Patients With Large Vessel Occlusion: A Case Series and Pooled Analysis
Source: Front Neurol. 2020 Aug 27;11:924. doi: 10.3389/fneur.2020.00924 (PMC7481477; doi:10.3389/fneur.2020.00924)
Supplement: Supplementary file 1 [file Table_1.DOCX]

**Table S1**. Clinical profiles on Y-stent thrombectomy in patients with emergent large vessel occlusion at different locations

| Case | Occlusion Location | Thrombus origin | Y-stent （Type/position） | Thrombectomy Procedure  （technique or stent retriever, pass） | Y-stent  AOL | Final eTICI | GTR (min) | Complication | mRS |
| --- | --- | --- | --- | --- | --- | --- | --- | --- | --- |
| 1 | L Carotid terminus | Embolic | Parallel / ACA + MCA | Solitaire FR 6ⅹ30 mm, 3;  Solitaire FR 6ⅹ30 mm + Revive 4.5ⅹ22 mm, 1 | 3 | 2b | 70 | PH | 6 |
| 2 | L Carotid terminus | Embolic | Parallel/ ACA + MCA | Solitaire FR 6ⅹ30 mm, 3;  Solitaire FR 6ⅹ30 mm + Revive 4.5ⅹ22 mm, 1 | 3 | 2b | 58 | None | 0 |
| 3 | L Carotid terminus | Embolic | Parallel/ ACA + MCA | Revive 4.5ⅹ22 mm, 2;  Solitaire FR 6ⅹ30 mm + Revive 4.5ⅹ22 mm, 1；  Solitaire 6 x 30mm，1 | 3 | 3 | 76 | Vasospasm | 0 |
| 4 | L Carotid terminus | Embolic | Parallel/ ACA + MCA | Solitaire FR 6ⅹ30 mm, 4;  Solitaire FR 6ⅹ30 mm + Revive 4.5ⅹ22 mm, 1；  Revive 4.5 x 22 mm，1 | 3 | 2b | 163 | None | 6 |
| 5 | L Carotid terminus | Embolic | Parallel/ ACA + MCA | Solitaire FR 6ⅹ30mm, 2;  Solitaire FR 6ⅹ30mm + Solitaire FR 4ⅹ20 mm, 1; Solitaire FR 4ⅹ20 mm, 2 | 3 | 2b | 196 | None | 5 |
| 6 | R Carotid terminus | Embolic | Parallel/ ACA + MCA | Solitaire FR 6ⅹ30 mm, 2;  Solitaire FR 6ⅹ30 mm + Revive 4.5ⅹ22 mm, 1; | 3 | 2b | 189 | None | 5 |
| 7 | R Carotid terminus | Embolic | Parallel/ ACA + MCA | ADAPT, 2; Solitaire 6 x 30 mm, 2;  Solitaire FR 6ⅹ30 mm + Revive 4.5ⅹ22 mm, 1;  Revive 4.5ⅹ22 mm, 1 | 3 | 2b | 124 | None | 1 |
| 8 | R Carotid terminus | Embolic | Parallel/ ACA + MCA | ADAPT，1;Solitaire FR 4ⅹ20 mm, 3; Solitaire FR 6ⅹ30 mm, 1; Solitaire FR 4ⅹ20mm + Solitaire FR 6ⅹ30 mm, 1; Solitaire FR 6ⅹ30 mm, 3; ADAPT, 1 | 2 | 2b | 235 | None | 0 |
| 9 | L Carotid terminus | Embolic | Parallel/ ACA + MCA | ADAPT，1; Revive 4.5ⅹ22 mm, 2; ADAPT, 1;;  Double Revive 4.5ⅹ22 mm, 1; ADAPT, 1;  Solitaire FR 6ⅹ30 mm, 2 | 2 | 2b | 66 | None | 2 |
| 10 | R Carotid terminus | Embolic | Parallel/ ACA + MCA | ADAPT, 3; Solitaire FR 4ⅹ20mm, 2; Solitaire FR 4ⅹ20mm + Aperio 3.5ⅹ20mm, 1 | 3 | 2a | 120 | SAH | 4 |
| 11 | R Carotid terminus | Embolic | Parallel/ ACA + MCA | ADAPT,1; Solitaire FR 6ⅹ 30mm, 5; Solitaire FR 6ⅹ 30mm + Revive 4.5ⅹ22 mm, 1；LVIS, 4.5ⅹ30 mm | 3 | 3 | 309 | None | 4 |
| 12 | L Carotid terminus | Embolic | Crossing / ACA + MCA | ADAPT，3；Solitaire FR 6ⅹ 30mm, 2；Solitaire FR 6ⅹ 30mm + Revive 4.5ⅹ22 mm, 2；Solitaire FR 6ⅹ 30mm, 1 | 0 | 0 | Fail | None | 5 |
| 13 | L Carotid terminus | Atherosclerotic | Parallel/ACA + MCA | Revive 4.5ⅹ22 mm, 3;  Solitaire FR 4ⅹ20 mm + Revive 4.5ⅹ22 mm, 1; Solitaire 4 x 20mm, 1 | 3 | 2a | 107 | Distal embolism | 4 |
| 14 | L Carotid terminus | Embolic | Parallel/ ACA + MCA | Solitaire FR 6ⅹ 30mm, 2；Solitaire FR 6ⅹ 30mm + Solitaire FR 4ⅹ20 mm, 1； | 3 | 3 | 44 | None | 4 |
| 15 | R Carotid siphon | Embolic | Parallel/ ICA + PComA | ADAPT,1; Revive 4.5ⅹ22 mm, 2;  Solitaire FR 4ⅹ20 mm+ Revive 4.5ⅹ22 mm, 1 | 3 | 2b | 62 | None | 1 |
| 16 | R Carotid siphon | Embolic | Parallel/ ICA + PComA | ADAPT,1; Solitaire FR 6ⅹ20 mm, 5;  Solitaire FR 6ⅹ30 mm+Solitaire FR 4ⅹ20 mm, 1；ADAPT, 1;  Solitaire FR 6ⅹ30 mm, 1； Apollo 6ⅹ13mm, stenting | 0 | 3 | 259 | None | 6 |
| 17 | R Carotid siphon | Embolic | Parallel/ ACA + MCA | ADAPT,1; Solitaire FR 6ⅹ30 mm, 3;  Solitaire FR 6ⅹ30 mm+ Solitaire FR 4 x 20 mm Y-stent，1；Solitaire FR 6 x 30 mm+ Revive 4.5 x 22mm, 1；LVIS 3.5ⅹ20mm stenting | 0 | 2b | 285 | None | 6 |
| 18 | R-MCA bifur. | Tandem lesion | Parallel/ superior and inferior trunk | Revive 4.5ⅹ22 mm, 2;  Solitaire FR 4ⅹ20 mm + Revive 4.5ⅹ22 mm, 2 | 1 | 1 | 180 | SAH | 4 |
| 19 | R-MCA bifur. | Tandem lesion | Parallel/ superior and inferior trun | Revive 4.5ⅹ22 mm, 3; Solitaire FR 4ⅹ20 mm, 1; Solitaire FR 4ⅹ20 mm + Revive 4.5ⅹ22 mm, 1；Solitaire FR 4 x 20 mm，1； Solitaire FR 4ⅹ20 mm + Revive 4.5ⅹ22 mm, 1 | 3 | 2b | 47 | None | 0 |
| 20 | L-MCA bifur. | Embolic | Parallel/ superior and inferior trunk | Revive 4.5ⅹ22 mm, 1; Solitaire FR 4ⅹ20 mm, 1; Solitaire FR 4ⅹ20 mm + Revive 4.5ⅹ22 mm, 1 | 3 | 3 | 61 | SAH | 4 |
| 21 | L-MCA bifur. | Embolic | Crossing / superior and inferior trunk | Solitaire FR 4ⅹ20mm, 2；Solitaire FR 6ⅹ30mm, 1；Solitaire FR 4ⅹ20mm+ Solitaire FR 6ⅹ30mm, 1 | 3 | 3 | 109 | None | 1 |
| 22 | Basilar terminus | Embolic | Parallel /Bilateral PCAs | Revive 4.5ⅹ22 mm, 3;  Double Revive 4.5ⅹ22 mm, 1 | 3 | 3 | 72 | None | 0 |
| 23 | Basilar terminus | Embolic | Parallel/ Bilateral PCAs | Solitaire FR 4ⅹ20mm, 3; Double Solitaire FR 4ⅹ20mm, 1; | 3 | 2b | 111 | None | 1 |
| 24 | Basilar terminus | Embolic | Parallel/ Bilateral PCAs | ADAPT, 2; Solitaire FR 4ⅹ20mm, 1; Solitaire FR 4ⅹ20mm +Revive 4.5ⅹ22mm, 1 | 3 | 2b | 100 | None | 1 |
| 25 | Basilar terminus | Tandem lesion | Parallel/ Bilateral PCAs | Solitaire FR 4ⅹ20mm, 2；double Solitaire FR 4ⅹ20mm, 1 | 3 | 3 | 147 | None | 0 |
| 26 | Basilar terminus | Embolic | Parallel/ Bilateral PCAs | Trevo 4ⅹ20mm, 2; Solitaire FR 4ⅹ20mm, 1; Trevo 4ⅹ20mm + solitaire FR 4ⅹ20mm, 3; Solitaire FR detachment | 3 | 2b | 227 | Dissection | 5 |
| 27 | Basilar terminus | Embolic | Crossing /Bilateral PCAs | Solitaire FR 4ⅹ20mm, 2; Solitaire FR 4ⅹ20mm+ Solitaire FR 6ⅹ30mm, 1 | 3 | 3 | 71 | None | 6 |
| 28 | Basilar terminus | Embolic | Parallel/ Bilateral PCAs | Solitaire FR 4ⅹ20mm, 2; double Solitaire FR 4ⅹ20mm, 1; | 3 | 2b | 183 | None | 6 |

ICA, internal carotid artery; PCom.A, posterior communicating artery; T, terminus; bifur., bifurcation; MCA, middle cerebral artery; mRS, modified Rankin score; mTICI, modified thrombolysis in cerebral infarction; NIHSS, National Institutes of Health Stroke Scale; AOL, arterial occlusive lesion; GTR, groin puncture to recanalization time; ADAPT, a direct aspiration first-pass technique; SAH, subarachnoid hemorrhage; PH, parenchymal hematoma.
